# Sex and age estimation from cardiac signals captured via radar using data augmentation and deep learning: a privacy concern

**DOI:** 10.3389/fdgth.2025.1616770

**Published:** 2025-11-14

**Authors:** Daniel Foronda-Pascual, Carmen Camara, Pedro Peris-Lopez

**Affiliations:** Department of Computer Science, Carlos III University of Madrid, Madrid, Spain

**Keywords:** data augmentation, deep learning, radar-based cardiac signal, privacy, sex and age estimation

## Abstract

**Introduction:**

Electrocardiograms (ECGs) have long served as the standard method for cardiac monitoring. While ECGs are highly accurate and widely validated, they require direct skin contact, are sensitive to motion artifacts, and are not always practical for continuous or unobtrusive monitoring, limiting their generalization to real-world, dynamic environments. However, radar-based technologies offer a novel, non-invasive alternative for acquiring cardiac signals without direct contact. This improves both hygiene and patient comfort, making it especially attractive for medical applications. Despite these benefits, it may raise privacy concerns, inadvertently revealing personal attributes such as sex and age. This study investigates, for the first time, how such demographic information can be inferred from radar-acquired cardiac signals.

**Methods:**

To address this question, we developed a machine learning framework to predict demographic attributes from radar-based cardiac signals. These signals were transformed into scalograms—a time-frequency representation—and then classified using a Convolutional Neural Network (CNN). Given the lack of prior studies on demographic inference from radar-based cardiac signals, the generalization capabilities of existing approaches remain untested in this context. Moreover, the small size of available datasets further limits model performance. To mitigate these issues, we applied data augmentation using a Conditional Wasserstein Generative Adversarial Network (cWGAN), which generated synthetic scalograms conditioned on class labels. Notably, there are very few prior studies on data augmentation specifically for this type of signal. This strategy aimed to enhance model accuracy and generalization by enriching the training data.

**Results:**

Our experiments demonstrate that data augmentation significantly improves model performance. The trained model achieved an accuracy of 78.40% in predicting the sex of individuals and 72.83% accuracy in classifying them into two age groups (18–29 and 30–65 years), despite the dataset being limited to only 30 subjects.

**Discussion:**

These findings reveal a potential privacy risk associated with radar-based biometric systems. The ability to infer sensitive demographic information from physiological signals could have serious implications, particularly in secure applications such as electronic passports (e-passports), where access to RFID chip data often depends on such personal attributes. Therefore, while radar technologies offer promising advantages, their deployment must consider and address the associated privacy challenges.

## 1 Introduction

The significance of biosignals in contemporary society, particularly within the healthcare system, has grown steadily in recent years. This trend is driven by advancements in technology, including the development of sophisticated sensors and wearable devices or data capture through the Internet of Things. These innovations allow for continuous and real-time monitoring of patients, leading to early detection of anomalies and more effective management of chronic diseases. Furthermore, the application of artificial intelligence (AI) and machine learning algorithms to biosignal data enhances the accuracy of diagnoses and prognoses, paving the way for personalized medicine. Beyond traditional medical applications, biosignals are also being utilized in emerging fields such as human-computer interaction, neuroprosthetics, mental health monitoring, and security. The ability to interpret biosignals accurately is transforming how healthcare providers approach patient care, emphasizing preventive measures and improving overall health outcomes.

Biosignals play a crucial role in various contemporary applications. Among the most common, electrocardiograms (ECGs) record the heart’s electrical activity, essential for diagnosing cardiac diseases. Electroencephalograms (EEGs) capture brain activity, aiding in neurological diagnoses. Electromyograms (EMGs) measure electrical impulses in muscles, assessing muscle function and diagnosing neuromuscular conditions. Other biosignals, such as galvanic skin responses (GSRs), measure changes in skin conductance due to emotional or physiological arousal. Respiratory signals, along with blood pressure and oxygen saturation (SpO2) measurements, are fundamental in monitoring cardiovascular and respiratory health [1].

Within these signals, cardiac monitoring is essential in modern healthcare. Specifically ECGs are instrumental in diagnosing various cardiac conditions, including arrhythmias, myocardial infarctions, and heart rhythm abnormalities. By capturing the depolarization and repolarization of cardiac muscle cells, ECGs provide critical insights into heart function and health. In clinical practice, ECGs are obtained through electrodes placed on the skin, typically on the chest, limbs, and sometimes the torso. These electrodes detect the tiny electrical impulses generated by the heart with each heartbeat. The resulting waveform reflects different phases of the cardiac cycle and abnormalities in these patterns can indicate specific cardiac disorders. Beyond diagnosis, ECGs are also widely used in research to study cardiac function under various conditions and interventions. Additionally, they have proven valuable in fields such as emotion recognition [2], and in security applications, where they serve as a potential biometric identifier due to their unique characteristics and the possibility of continuous authentication without requiring user cooperation [3, 4]. Additionally, research in fields such as human-computer interaction and wearable technology uses cardiac signals to improve user experience, monitor stress levels, and optimize performance.

However, obtaining an ECG always requires physical contact, which presents several inherent limitations. Firstly, contact-based methods such as ECG require specialized equipment for signal acquisition, which can be cumbersome and restrict accessibility and portability. Moreover, direct physical contact with the user may raise concerns regarding comfort, hygiene, and potential skin issues, especially in sensitive populations like premature babies undergoing continuous monitoring [5]. Furthermore, these devices are susceptible to movement artifacts, which can compromise measurement accuracy, particularly in dynamic environments. In contrast, contactless methods using radar technology provide an interesting alternative. Doppler radar operates by emitting electromagnetic waves towards the chest area, where they interact with the surface of the skin and underlying tissues. As the heart beats, it causes subtle movements in the chest surface. These movements alter the frequency of the reflected waves due to the Doppler effect, where the frequency shift is directly related to the velocity of the chest movements induced by the heartbeat. By analyzing these frequency shifts over time, Doppler radar can extract detailed information about cardiac activity, including heart rate and rhythm enabling non-intrusive cardiac motion detection and continuous monitoring without the need for physical contact [6]. This approach enhances user comfort and convenience; however, it currently faces some limitations. One of them is the susceptibility of radar signals to noise and interference from various environmental sources, which can compromise the reliability of measurements. As a result, achieving clinical-grade accuracy remains a ongoing challenge, particularly in real-world scenarios where uncontrolled environmental factors and patient mobility can affect the precision of radar-based health monitoring. Furthermore, there are significant challenges in radar signal processing and the application of machine learning, especially with advanced deep learning techniques. Researchers are focused on identifying the most effective methods for extracting vital information from radar data and analyzing it robustly, ensuring functionality across a wide range of situations [7].

However, embedded within these previously described biosignals are implicit personal details, which may inadvertently disclose sensitive information about individuals like sex or age. While this data are essential for medical insights, their unintended disclosure poses significant privacy risks. Unauthorized access or misuse of such information could lead to discrimination, compromised personal privacy, or even targeted exploitation in various contexts [8], highlighting the importance of robust data protection measures in biosignal processing and storage. According to major privacy regulations such as the General Data Protection Regulation (GDPR) [9] in Europe and the California Consumer Privacy Act (CCPA) [10] in the United States, personal information includes not only direct identifiers but also data that can be reasonably linked to an individual, including inferred attributes used for profiling. There are also concrete security implications: for example, sex and age are used in deriving access keys for RFID chips embedded in e-passports, so their unauthorized inference via remote sensing could reduce key entropy and facilitate brute-force attacks [11]. Furthermore, such inferred data can be exploited to segment individuals into demographic or consumer groups, potentially enabling manipulative advertising, discriminatory pricing, or other unauthorized profiling practices, as specified in Section (K) of the CCPA. The potential leakage of personal information, alongside other medically relevant data, has been explored in several biometric modalities, including ECG [12], EEG [13], photoplethysmography (PPG) [14, 15], and gait signatures [16]. These studies demonstrate the unintended disclosure of sensitive attributes, such as age and sex. However, to the best of our knowledge, no comprehensive investigation has yet addressed this concern for cardiac signals derived from radar technology, representing a significant gap in the study of this type of signal.

The mechanism behind this potential privacy leakage lies in the inherent encoding of demographic information within the morphological and spectral features of cardiac signals. For instance, differences in chest anatomy, hormonal influences on heart rate variability, or vascular compliance associated with aging can subtly alter the frequency components and temporal structure of the radar-extracted signal. When processed using advanced machine learning models, these patterns can be exploited to infer attributes such as sex and age—even if such information was not explicitly targeted. This raises significant privacy concerns, especially in security-sensitive environments such as biometric authentication systems or electronic passports. Technically, radar-based monitoring still faces challenges such as noise susceptibility, difficulty in isolating the cardiac component from respiration and motion artifacts, and a lack of large, labeled datasets—factors that complicate the generalization and reliability of deep learning models trained on this data. Furthermore, traditional signal processing approaches often fall short in capturing the complex time-frequency dynamics needed to robustly infer demographic features, motivating the need for deep learning and data augmentation solutions. These challenges and opportunities motivate the present study, which aims to systematically investigate the presence and implications of such demographic leakage.

The objective of this article is to investigate whether this information (sex and age) is implicitly encoded in cardiac signals extracted via radar. In other words, our main question is: Is there a leakage of age and sex, along with other medical attributes, in the heart signal extracted from radar? To this end, we aim to estimate both variables from this type of signal. Understanding this potential correlation could provide insights into novel applications and implications for biometric identification and motivate the exploration and development of different security methods that could improve authentication and privacy protection.

The key contributions of this paper are as follows:

1. We present the first study that demonstrates the leakage of demographic information (sex and age) from radar-acquired cardiac signals. While prior works have explored this in ECG and PPG signals, no such analysis has been conducted using radar technology. Our study addresses this gap and provides empirical evidence supporting the presence of demographic markers in radar signals.
2. We propose a novel data augmentation approach based on Conditional Wasserstein GANs (cWGAN), specifically tailored to scalograms of radar-extracted heartbeat signals. Unlike previous methods that rely on ECG as a generative input, our approach directly synthesizes radar-based scalograms conditioned on class labels, enabling better balance and generalization in small datasets.
3. Our experiments show that the use of cWGAN-generated scalograms significantly improves classification performance. For sex classification, accuracy increases from 72.80% to 78.40% (+5.6%), and for age group classification from 65.60% to 72.83% (+7.23%). Frame-level accuracy, FAR, FRR, precision, recall, and F1-score all show consistent improvements with data augmentation.
4. We ensure robust evaluation using leave-one-subject-out cross-validation, preventing data leakage and simulating realistic scenarios where the model must generalize to unseen individuals.
5. We highlight the privacy implications of our findings, particularly in biometric applications where demographic leakage from cardiac signals could compromise personal security.

The rest of this article is structured as follows. The Related Work section reviews the existing scientific literature on the use of radar-based heart signals, age and sex estimation through biosignals, and data augmentation in this field. The Materials and Methods section provides detailed information about the dataset, preprocessing steps, and the methodology used for age group and sex estimation with radar data. It also explains the data augmentation techniques employed to enhance the training dataset. The Results section presents the study’s findings, evaluating the performance of the proposed methods using various metrics. Finally, the article concludes with a discussion of the results and their implications for future research.

## 2 Related work

### 2.1 Radar-based heart signal applications

Within the field of cardiac monitoring, one of the most widely utilized signals is the ECG, which finds application across various domains such as clinical monitoring [17–19], diagnosis of cardiac conditions [20–23], sports medicine [24], stress monitoring [25], emotion recognition [2, 26] or biometric identification [27, 28].

Non-invasive health monitoring solutions, such as radar-based systems, are emerging as alternatives to traditional methods like PPG and ECG, which generally require contact-based sensors [29]. These non-contact radar technologies allow for continuous and unobtrusive physiological monitoring, offering a comfortable solution for patients in both clinical and everyday environments. With applications ranging from vital sign detection [30] and respiratory monitoring [31] to blood pressure estimation [32] and cardiac monitoring [33], radar systems represent a significant advancement in the context of non-invasive health technology. However, despite the advancements in non-contact monitoring, research on radar-based cardiac signal analysis remains limited. A promising approach within this field is the use of Microwave Doppler sensors, which enable the extraction of heartbeat and individual feature quantities through time-frequency analysis without requiring direct skin contact. These sensors are sensitive enough to detect minute vibrations on the chest surface caused by heartbeats after isolating these vibrations from other movements such as respiration or artifacts. To address this issue, some studies [34, 35] employ Butterworth filters to attenuate lower frequencies associated with respiration. Conversely, [36] proposes Wavelet Packet Decomposition (WPD) as an alternative method, achieving less than 2% error for respiration and 3.5% for heart rate, thereby enhancing the accuracy of vital sign detection compared to Bandpass filters and Peak Detection methods. Furthermore, in [37], various techniques were assessed to identify the most effective approach for extracting cardiac signals from radar data, with Discrete Wavelet Transform (DWT) demonstrating superior performance, even surpassing WPD.

Many studies that work with this type of radar-obtained signal use Machine Learning techniques to extract information [38]. Applications can be quite diverse, such as estimating the patient’s heart rate [39–43] or heart rate variability [44–46], with some studies also applying this process to animals [47–49]. In [50] they use commodity Wi-Fi devices to capture the heart signal of subjects, and in [51] the heart signal of different subjects who are simultaneously in the same environment is captured using radar. In addition to detecting heart rate, work has also been done on the detection or monitoring of breath rate, as in [52] or more generally reviewed in [53]. Another field that has received much attention is the monitoring of both heartbeats [54–58] and vital signs [59–61]. The possible applications of these types of signals in security have also begun to be investigated, for example, with the aim of authenticating people in a convenient, contactless, and continuous manner although the results are still scarce. In [62], they create an authentication method based on the fiduciary analysis of the heart signal (which involves using specific reference points in the signal as references). In [63], they perform authentication by separating the signal into heartbeats and classifying each one, while in [64], they transform the signal into a scalogram to then extract features and classify using Deep Convolutional Neural Networks (DCNNs), achieving an accuracy of 98.5% in a sample composed of only 4 people. In [65], they attempt identification in a set of 20 subjects using the spectral distribution of the signal and obtain an Error Rate (EER) of 3.48%. On the other hand, the use of these types of signals for other purposes has been explored, such as identification from respiratory signals [66], gait recognition [67], event recognition [68], emotion recognition [69], and multiperson spatial tracking [70]. The main limitation in the development of this area, however, is the lack of datasets that collect this type of signal. Currently, those that exist do not usually have a large number of subjects, which sometimes prevents presenting more solid results.

### 2.2 Age and sex estimation

The field where estimation of age and sex has been most extensively researched is through facial images [71]. However, there is also notable work using other signals such as ECG [12].

Various methods have been employed to estimate sex from ECG, with the use of CNNs being among the most frequent approaches. This is demonstrated in [72] achieving 90.4% accuracy. In [73], clustering of heartbeats into 10 groups followed by training separate classifiers for each group aimed to maximize performance, achieving 94.4% accuracy. On the other hand, estimated age from ECG reflects the overall health status of the cardiovascular system. When significantly above chronological age, it may indicate the presence of abnormalities or disorders [12]. In [74], they attempt to predict sex and age group (young/old) from ECG using CNNs, achieving accuracies of 86.82% for sex and 82.97% for age group. Most current studies utilize Deep Learning to estimate cardiac age, employing CNNs with ECG signals as network inputs, as demonstrated in [75] or in [72] where an average error of 6.9 ± 5.6 years in age estimation was reported. In [76], a neural network regression model was used to predict chronological age from ECG with the same mean error of 6.9 ± 5.6 years, while in [77], a Deep Learning model achieved a mean error of 6.899 years. The advantage of estimating age and sex from ECG lies in the availability and size of datasets, sometimes exceeding 700,000 patients as seen in [72].

However, when it comes to estimating age and sex from radar-extracted cardiac signals, there is limited research. [78] estimates sex based on the act of transitioning from sitting to standing, and [79] estimates it using gait features, both studies using radar to capture body movement rather than heartbeats. Similarly, [80] uses radar to assess body movement in elderly individuals to estimate their age and fall risk. In [81], authors do study sex differences in relation to radar-detected cardiac signals, finding that the reference signal-to-noise ratio is significantly higher in males than females, possibly due to chest anatomy differences, making it somewhat more challenging to detect cardiac signals with this technique in females compared to males. Therefore, to the best of our knowledge, no prior studies have attempted to estimate sex or age from radar-detected cardiac signals.

### 2.3 Data augmentation

Data augmentation is a technique used to improve the performance of machine learning and deep learning models by artificially expanding the size and diversity of training datasets. In the context of biosignals, this can be particularly beneficial as datasets are often limited due to challenges in collecting large amounts of certain types of medical data. However, given the wide variability among classes of these data, data augmentation techniques can vary significantly depending on the biosignal being studied.

When working with ECG data, studies often employ data augmentation techniques that rely on signal transformations or a combination thereof, rather than utilizing deep learning methods. For instance, in [82], researchers develop an algorithm that optimizes the order and parameters of these transformations. These methods include time masking, which zeros out parts of the signal, SpecAugment, which masks components in both the time and frequency domains of a signal transformed using Short-Time Fourier Transform (STFT), Discriminative Guided Warping, using Dynamic Time Warping to align a source ECG with a representative reference signal distinct from other classes, and SMOTE, an oversampling strategy that generates synthetic examples of the minority class by interpolating minority samples to address class imbalance in ECG prediction problems. Similarly, studies like [83–85] employ a combination of transformations to augment the training set, whereas [86] augments heartbeats specifically to mitigate class imbalance within their dataset.

A common scenario when applying data augmentation techniques is to work with images as inputs, for which there are multiple strategies to increase the training data, among which the use of GANs is one of the main ones [87]. This situation is frequently encountered when working with EEG, where different data augmentation techniques can yield good results [88]. In [89] new samples are obtained after training a GAN to regenerate a part of the image that has been erased, demonstrating that this method can improve emotion recognition. In [90], they use a similar procedure but employing a Conditional Wasserstein GAN (cWGAN), also improving the results in emotion classification, just like in [91], where they use a multiple generator cWGAN for the same purpose. In [92], a conditional Boundary Equilibrium GAN is employed to generate new samples of EEG data, eye movement data, and their direct concatenations. Meanwhile, in [93], GANs are utilized to augment one-dimensional data. On the other hand, [94] demonstrates the use of a Wasserstein GAN to enhance Ground-penetrating radar data, achieving superior results compared to auxiliary classifier GANs and other traditional methods. Finally, in [95], a Wasserstein GAN with confidence loss is applied to augment a dataset of plant images. Similarly, [96] presents an alternative strategy for augmenting ECG signals based on segmentation and rearrangement of time-series data. This method, although not based on deep generative models, allows the generation of realistic and well-structured synthetic signals with minimal computational cost. Another recent approach [97] focuses on emotion recognition from ECG signals using a unimodal system. Given the scarcity of labeled affective ECG datasets, the authors propose a multi-filtering augmentation method, which generates new data by applying diverse filtering techniques to enhance signal quality showing a significant performance boost.

Wasserstein GANs (WGANs) are a type of GAN that utilize the Wasserstein distance, also known as Earth Mover’s Distance (EMD), as a loss function to improve the training stability and performance of GANs [98]. Traditional GANs often suffer from issues such as mode collapse and vanishing gradients, which can make training difficult and unstable. The Wasserstein distance provides a smoother and more meaningful measure of the difference between the generated data distribution and the real data distribution, helping to mitigate these issues. Gradient penalty and spectral norm are two enhancements used to enforce the Lipschitz constraint in WGANs, further improving training stability. Gradient penalty adds a penalty term to the loss function to constrain the gradient norm of the critic’s output with respect to its input to be close to 1. This is done by interpolating between real and generated data points and penalizing deviations from this gradient norm, with the penalty term typically calculated as $\lambda \cdot (\|\nabla_{\hat{x}}D(\hat{x}){\|}_{2}-1{)}^{2}$, where $\hat{x}$ are interpolated samples, $D(\hat{x})$ is the critic’s output, and λ is a coefficient determining the penalty strength. Spectral norm, on the other hand, normalizes the weight matrices of the neural network layers by their spectral norm, which is the largest singular value of the weight matrix. By constraining the spectral norm to be at most 1, the Lipschitz continuity of the critic function is ensured without the need for weight clipping or gradient penalties, making training more stable and reducing the risk of exploding or vanishing gradients.

An example of this technique is used in the study [99], where researchers working with accelerometer data for human activity recognition demonstrated that augmenting training data using cWGAN is more effective than using a conditional GAN. Regarding data augmentation for heart signals obtained through radar technology, we found only two studies. In [100] they use an attention-based WGAN with Gradient penalty to generate new I/Q signals, feeding the generator with ECGs. The objective is to classify heart signals into two groups: low heart rate (less than 60 beats per minute) or high. In [101], the authors utilize the decomposition provided by the Discrete Wavelet Transform to augment the data. However, they train the model with the newly created samples and test it on the original samples, which may lead to potential data leakage.

In summary, while previous research has shown the feasibility of using radar signals for vital sign detection and even biometric tasks, there is a lack of studies focused on privacy risks—specifically the unintended leakage of demographic information such as age and sex. Moreover, existing works often rely on small datasets and do not leverage advanced data augmentation techniques tailored to this signal type. Most GAN-based approaches in biosignal processing either require complementary signals (e.g., ECG) or are not designed for conditional generation of demographic categories. These gaps motivate our proposed method, which directly addresses the challenges of limited data, signal ambiguity, and demographic inference through an end-to-end deep learning and data augmentation pipeline.

## 3 Materials and methods

**Figure 1:**
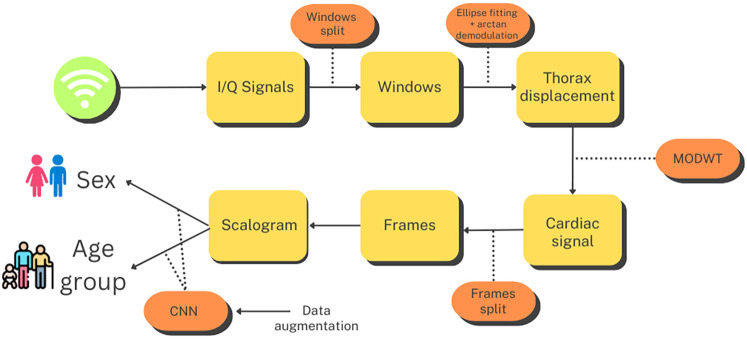
Flowchart for the age group and sex estimation process. Created using Canva, licensed under Free Content License.

This section describes the methods employed throughout the study. A graphical summary of the workflow for this process is presented in Figure 1. The first step after capturing the I/Q quadrature signals was to divide them into 10 s windows. Subsequently, we performed preprocessing on each window, as detailed in Section 3.2, to extract a signal corresponding to thoracic movement. From this signal, we isolated the component related to the heartbeat using the Maximal Overlap Discrete Wavelet Transform (MODWT), eliminating the influence of respiration and other noise. Next, we subdivided the 10 s heartbeat signal into overlapping frames of 4 s each and generated a scalogram for each frame. These scalograms, along with additional scalograms artificially generated using a cWGAN, were then used to train a CNN that classified each scalogram into male or female categories or age groups. Finally, to derive a single prediction for each 10 s window, we conducted a voting process based on the predictions provided by the CNN for each one of its frames.

### 3.1 Data

**Figure 2:**
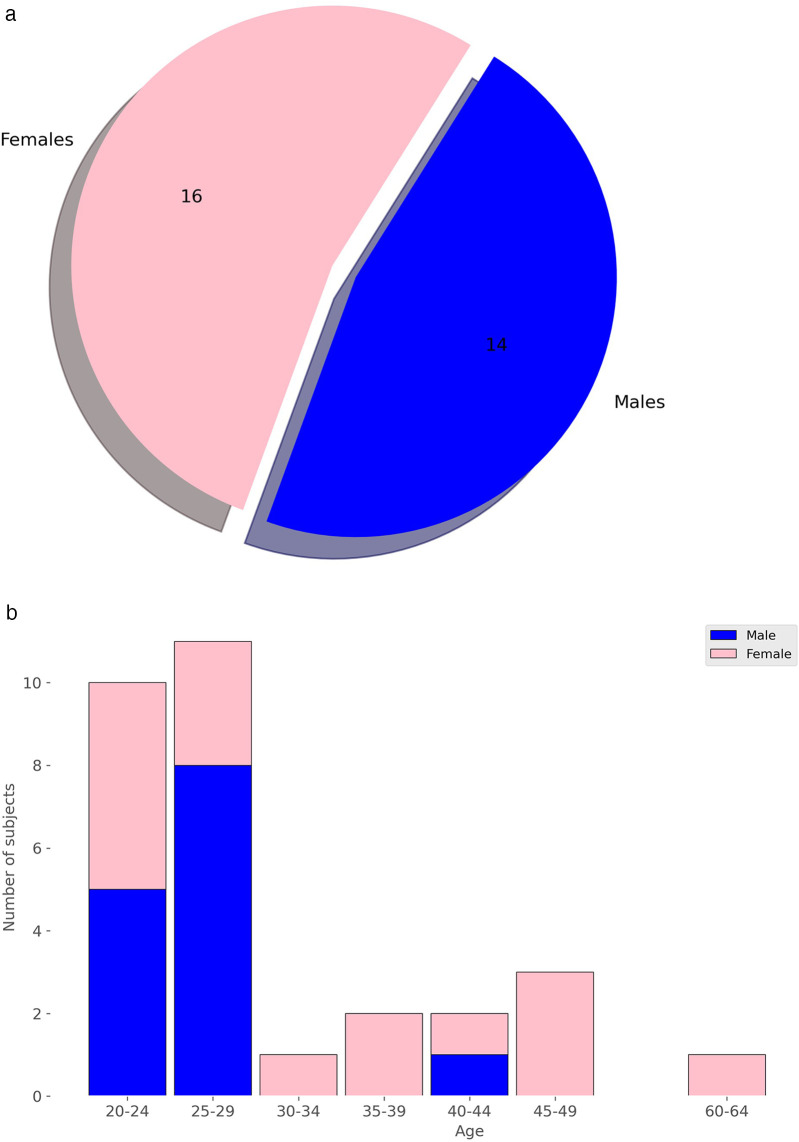
Distribution of subjects by sex and age. **(a)** Distribution by Sex. **(b)** Distribution by Age.

The study utilized a publicly available dataset from [102], collected at University Hospital Erlangen, Germany, involving 30 healthy participants (14 males, 16 females, with an average age of 30.7 years). A radar system, optimized with a focal point at approximately 40 cm, employed Six-Port technology to capture chest movements in a contactless manner. The system featured a bi-static antenna setup with transmitting and receiving antennas set at $\pm 10^{\circ }$ angles and a laser for alignment purposes. The dataset included recordings from five distinct scenarios: a resting scenario, Valsalva maneuver, apnea, tilt-up, and tilt-down. In this study, we only used recordings from the resting scenario, where participants reclined for at least 10 min in a relaxed position with calm breathing. The data consisted of In-Phase and Quadrature (I/Q) signals sampled at 2,000 Hz from the 30 different recordings, each approximately 10 min long. Figures 2a and 2b show the distribution of subjects by sex and age. Due to the highly imbalanced age data, we divided ages into two groups: 18 to 29 years and 30 to 65 years. The dataset is accessible at [103].

Although the dataset used in this study comprised only 30 individuals, this sample size was comparatively large within the current state of radar-based cardiac signal research. As reviewed in [104], most datasets incorporating radar signals—both public and private—were significantly smaller, with a median of just 12 subjects and an interquartile range of 5 to 24. Among the few public datasets available (only 10 in total), only one included more subjects than ours; however, it contained only children under the age of 13, making it unsuitable for our study. Therefore, the field of radar-based cardiac signal analysis remains in an early stage, and datasets of a size comparable to those available for other signals—such as ECG or PPG—are not yet available. Consequently, our use of 30 adult participants represents a substantially above-average sample size in this emerging research domain and marks a meaningful step forward in demonstrating the feasibility of demographic attribute estimation from radar signals at a scale larger than typically seen in previous work. Furthermore, we ensured strict subject-wise separation between training and testing data to avoid data leakage and to enhance the generalizability of our results. It is also reasonable to assume that, with larger datasets encompassing a broader diversity of subjects and recording conditions, the model’s generalization capability would further improve, potentially leading to even higher classification performance. This design strengthens the validity of our findings and provides a valuable contribution to a field still constrained by limited data availability.

### 3.2 Preprocessing

The raw cardiac signals used as input to our models consisted of I/Q components. These complex-valued signals were recorded at a sampling frequency of 2,000 Hz and captured subtle chest movements in a contactless manner. Using these I/Q signals provided by the dataset, the initial step involved segmenting them into non-overlapping 10 s windows, a window size that aligns with common practices in the state-of-the-art [105]. Subsequently, ellipse fitting was applied to the I/Q data points within each window, following the methodology outlined in [106]. This process enables arc tangent demodulation using the parameters derived from the fitted ellipses [107]. The outcome of this procedure represents the movement observed in the region of interest (the thorax), which encompasses various components, notably including the patient’s movements, respiratory patterns, and cardiac motion. To isolate the cardiac motion signal, we employed the Maximal Overlap Discrete Wavelet Transform (MODWT). MODWT is particularly effective in this context due to its ability to perform multiresolution analysis without the downsampling step present in classical discrete wavelet transforms (DWT). This property ensures translation invariance, which is essential when time alignment of features such as heartbeats must be preserved across frames. Radar signals from the thorax are highly non-stationary and contain overlapping frequency components from respiration, subtle body movements, and cardiac activity. MODWT offers a data-driven decomposition that adaptively separates these sources by their dominant frequency ranges, outperforming traditional bandpass filters. In [37], this approach has been evaluated against alternative methods, demonstrating superior performance. Subsequently, the cardiac motion signal within each window was segmented into overlapping 4 s frames with a stride of 0.5 s for classification, similar to the methodology outlined in [63] for heartbeat detection. Each frame was then transformed into its scalogram, represented as a black-and-white image with dimensions of 200×200 pixels. The CNN produces predictions at the frame level. To obtain the final decision for each 10 s window, frame-level probabilities are aggregated into a single probability distribution, from which the window-level prediction is derived. This voting scheme ensures more robust and stable classifications by combining evidence from multiple frames.

### 3.3 Data augmentation

In accordance with findings from other studies [108–110], we performed data augmentation using a GAN [111], specifically a cWGAN. This kind of WGAN incorporates conditional information: both the generator and the discriminator (or critic) receive additional input, typically in the form of class labels or other conditioning variables. The architectures of our generator and critic can be seen in Tables 1, 2 respectively. We used Adam as the optimizer for the generator, and RMSprop, as recommended in [98], for the critic. Additionally, we implemented gradient penalty with a coefficient of 10 to stabilize the training process, which helps enforce the Lipschitz constraint by ensuring the gradients have a norm close to 1. For both tasks, predicting sex and age respectively, each data sample was assigned a single label.

**Table 1:** Architecture summary of the generator in the cWGAN with 2 labels and 100 as input dimension.

Layer	Configuration	Activation	Other
1	ConvTranspose2d(102, 256, 3, 2)	ReLU	Batch normalization
2	ConvTranspose2d(256, 128, 3, 2)	ReLU	Batch normalization
3	ConvTranspose2d(128, 64, 5, 3)	ReLU	Batch normalization
4	ConvTranspose2d(64, 32, 3, 3)	ReLU	Batch normalization
5	ConvTranspose2d(32, 16, 3, 2)	Tanh	–

**Table 2:** Architecture summary of the critic for cWGAN with 2 labels.

Layer	Configuration	Activation	Other
1	Conv2d(1, 128, 4, 2)	LeakyReLU	Batch normalization
2	Conv2d(128, 256, 4, 2)	LeakyReLU	Batch normalization
3	Flatten()	–	–
4	Linear(1024 + 2, 64)	LeakyReLU	–
5	Dropout()	–	–
6	Linear(64, 1)	–	–

The cWGAN addressed the challenge of limited and imbalanced radar datasets by synthetically generating realistic scalograms conditioned on class labels (e.g., sex or age group). By modeling the data distribution and introducing controlled variability, the cWGAN enriched the training set with representative samples that improved generalization and reduced overfitting. This approach is particularly effective when collecting more real radar data is impractical due to time, cost, or ethical constraints. By training our cWGAN on the scalograms of patients dedicated to the training set, we were able to generate new scalograms labeled in each of the two categories: male and female in the case of sex, and 18–29 and 30–65 in the case of age. We chose this strategy instead of oversampling or undersampling to preserve the original distribution and avoid overfitting. Although other methods such as SMOTE or class weighting were considered, the cWGAN was selected for its ability to generate label-consistent and structurally realistic samples.

Our cWGAN was trained for 151 epochs with a batch size of 32. Early stopping was not used, but both generator and critic losses were monitored throughout training. The generator used a learning rate of $8\times 10^{-5}$, while the critic (or discriminator) used a smaller learning rate of $6.7\times 10^{-6}$. For every generator update, the critic was updated five times. The latent space dimension was set to 100. We conditioned the generation on two labels (e.g., male/female or age group), which were embedded and concatenated to both the generator input and the critic input. The training was carried out on scalograms corresponding to the training set, and a gradient penalty with coefficient 10 was applied to enforce the Lipschitz constraint and promote stable convergence.

### 3.4 Classification

We utilized a CNN for the classification task, as these models are frequently used in computer vision tasks, particularly as feature extractors from images across various fields [112, 113]. They concatenate hierarchical layers of convolutional filters to automatically learn meaningful representations from raw pixel data. These learned features capture hierarchical patterns such as edges, textures, and shapes, which are essential for understanding and classifying visual content. This feature extraction process from the scalograms is carried out in the convolutional layers of the CNN, while the fully connected layers placed at the end serve to classify the scalograms based on the previously obtained features.

**Figure 3:**
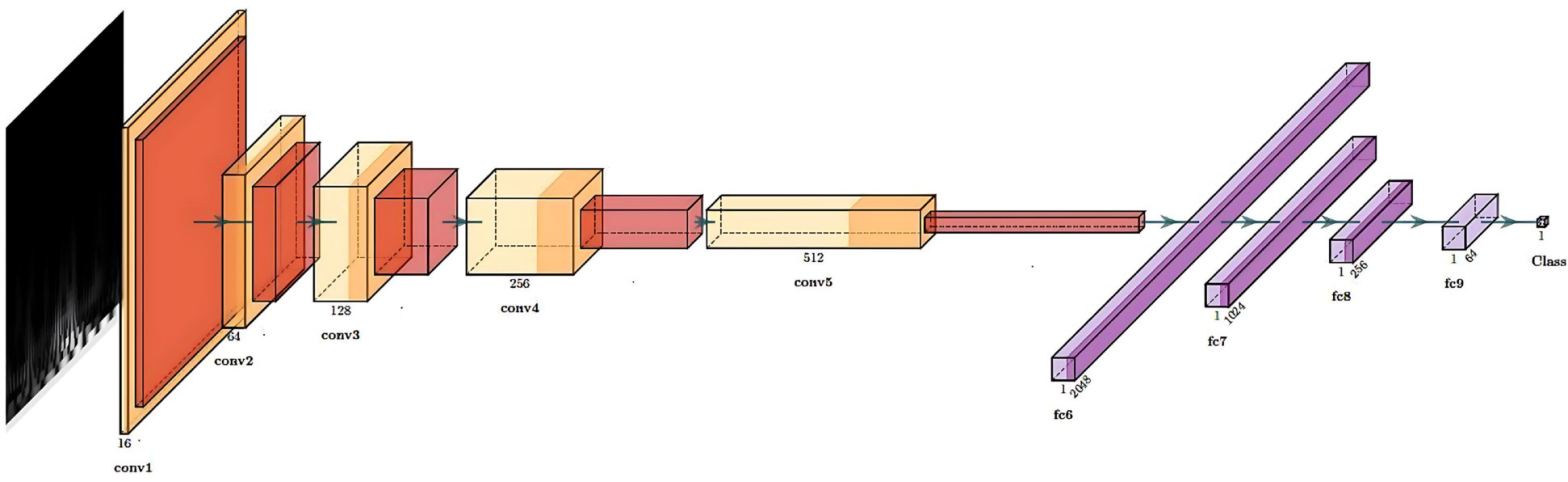
Structure of the CNN.

The structure of our CNN is shown in Figure 3 and consisted of 5 convolutional layers followed by 4 fully connected layers. The output was a number corresponding to the predicted class for the input scalogram. After each convolutional layer, we applied batch normalization to normalize the input and mitigate internal covariate shift [114]. The Rectified Linear Unit (ReLU) was used as the activation function to introduce non-linearity, which aids in learning complex patterns in the data. Max pooling was applied after each convolutional block to progressively reduce spatial dimensions and retain the most relevant features. The kernel sizes for the five convolutional layers were 5, 3, 13, 5, and 2 respectively, all with stride 1 and no padding. The input to the network consisted of 3-channel scalograms with fixed dimensions, and the size of the flattened feature map after the final convolutional block was 2048. The fully connected (MLP) part of the network contained layers of size 1024, 256, 64, and 1, with ReLU activations and dropout (*p* = 0.5) applied after each layer except the final output. The model was trained using the Adam optimizer with a learning rate of 0.001 and binary cross-entropy loss. With this model, we were able to predict the class of each scalogram, and thus of each frame. To obtain a prediction for each window, we then used hard voting based on the predictions of all the frames within that window. Nevertheless, we did not conduct an optimization process for the selected CNN architecture; therefore, alternative architectures might have yielded superior final results.

### 3.5 Privacy threat model

Radar-based cardiac sensing enables unobtrusive acquisition of physiological signals, often without requiring physical contact or active participation from the subject. While typically used for clinical monitoring or biometric authentication, this modality also allows for the potential inference of sensitive demographic attributes from the collected signals.

In our threat model, we considered a passive adversary who can covertly acquire radar reflections from a subject without requiring physical access to the sensing system. The adversary may place a radar device in the environment (e.g., in smart home devices, hospital rooms, or public kiosks) to continuously collect cardiac signals from individuals situated within sensing range. Data acquisition can occur in everyday scenarios where individuals are seated or stationary, and does not require user awareness or consent. The adversary is assumed to have access to trained deep learning models capable of inferring demographic attributes such as age or sex from radar-derived cardiac signals. By applying repeated predictions the adversary can aggregate results via majority voting to improve classification accuracy. Although individual predictions may be affected by noise, the accumulation of repeated inferences over extended periods enables reliable profiling of individuals based on latent features contained in the physiological signal.

### 3.6 Experimental setup

To prevent data leakage, training and testing sets were split at the subject level, ensuring that all frames from a given individual were assigned exclusively to either the training or the test set. Specifically, for each experiment, 80% of the subjects were randomly assigned to the training set and the remaining 20% to the test set, while maintaining a balanced distribution of sex or age groups as needed. Frames from the training subjects were used to train the CNN, including both real scalograms and synthetic scalograms generated by a cWGAN trained only on the training subjects’ data, while the test set contained only real scalograms. Consequently, neither the CNN nor the cWGAN was exposed to any data from the test subjects during training. This subject-wise splitting strategy guarantees that the CNN learns meaningful physiological features without any contamination from the test data.

All experiments were conducted on a workstation running Ubuntu 22.04.4 LTS. The system was equipped with a 12th Gen Intel Core i7-12700KF CPU running at 5.00 GHz, 32 GB of DDR4 RAM, and an NVIDIA GeForce RTX 3080 Ti GPU with 12 GB of VRAM. The code was executed using Python 3.11.7, PyTorch 2.3.0, and CUDA 12.2. For wavelet-based signal processing, the functions modwt and modwtmra from MATLAB’s Wavelet Toolbox were invoked via the Python-MATLAB engine. Key parts of the implementation, including model architecture, MODWT filtering, data generation with cWGAN and splitting functions will be made publicly available for full reproducibility [115].

## 4 Results

The aim of this article is to investigate whether personal data, specifically sex and age group, can inadvertently leak alongside other medically relevant information in cardiac signals obtained through radar technology. To address this issue, we will explore the feasibility of predicting these two variables from the radar-derived signals in a substantial number of cases. For the train-test split in the experiments, since the goal is to estimate two attributes specific to each person, age group and sex, we are separating the data by individuals. This ensures that signals from the same individual do not appear in both the training and testing sets, preventing possible data leakage, which may have occurred in the use of other biosignals [116].

### 4.1 Metrics

To evaluate whether cardiac signals obtained via radar transmit sensitive patient information, such as sex or age, in addition to heart-specific data, we will employ the following metrics in the classification process. Accuracy measures the overall effectiveness of the model by calculating the proportion of correctly classified instances out of the total. False Acceptance Rate (FAR) indicates how often unauthorized individuals are incorrectly accepted by the system, reflecting the rate of false positives among all actual negatives. On the other hand, False Rejection Rate (FRR) measures how frequently authorized individuals are incorrectly rejected, showing the rate of false negatives among all actual positives. Precision evaluates the proportion of true positive predictions among all positive predictions made by the model, while recall assesses the proportion of true positive instances correctly identified out of all actual positives. Finally, the F1-score combines precision and recall into a single metric, offering a balanced measure of the model’s performance in identifying positive instances [117].

### 4.2 Sex

**Figure 4:**
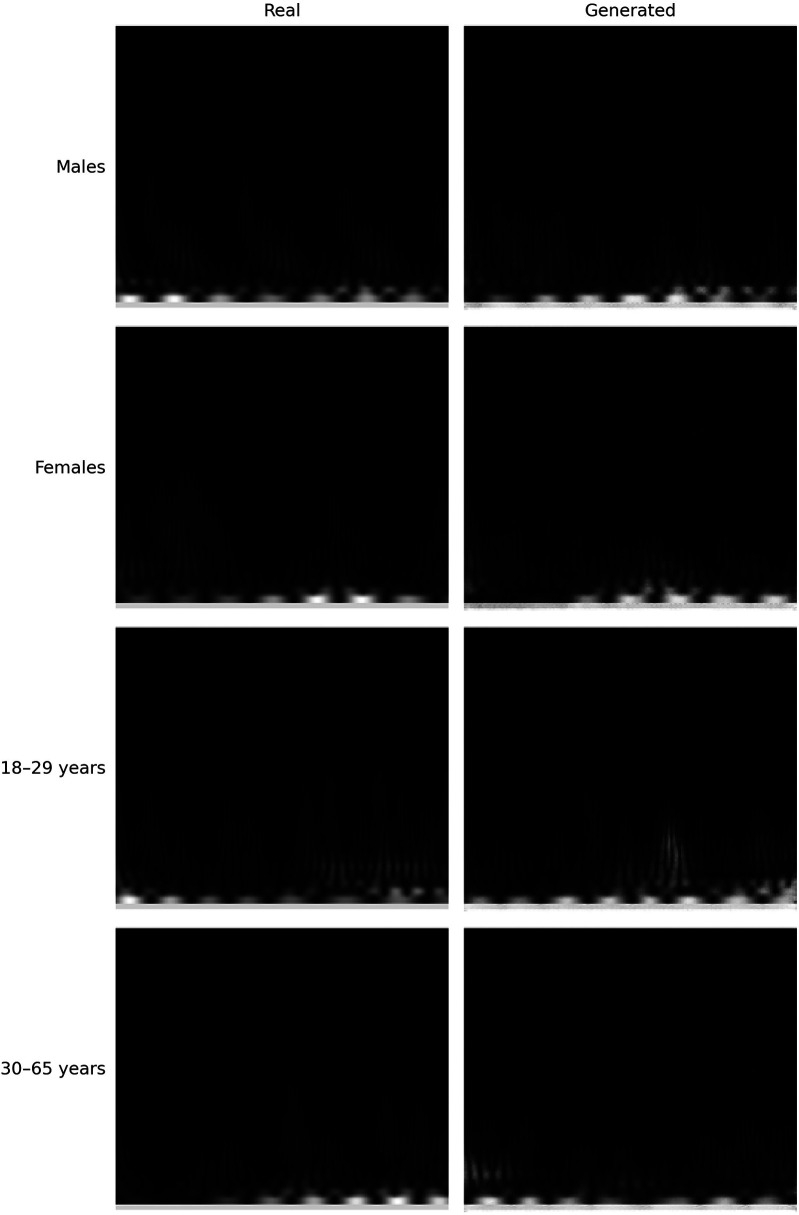
Comparison between real and synthetic scalograms generated by the cWGAN.

**Figure 5:**
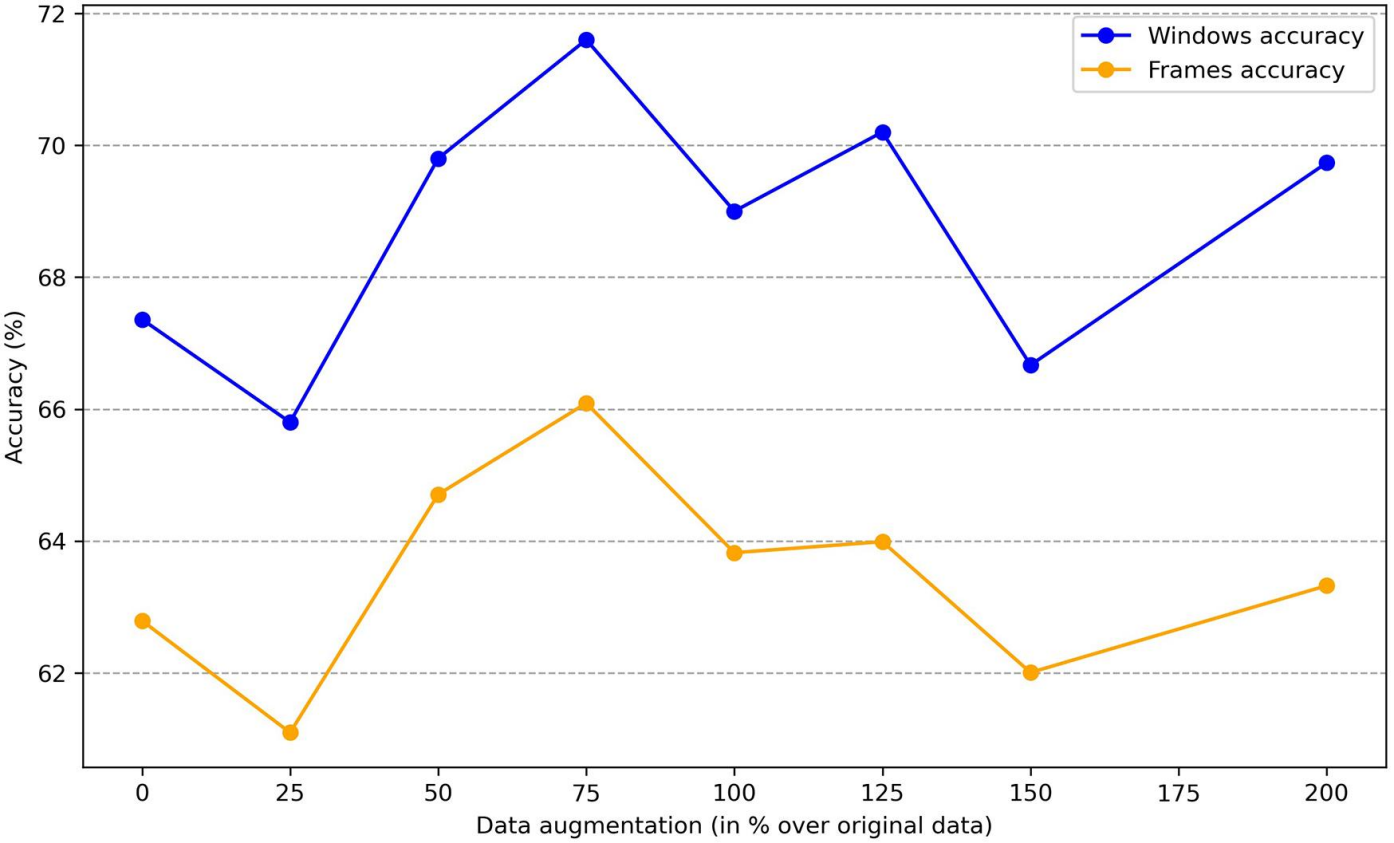
Average accuracy on sex estimation by data augmentation size.

To determine whether sex information is being inadvertently transmitted through the cardiac signals, we attempted to predict the sex of patients set aside for testing by training the classification model on the remaining ones. The dataset consists of 30 patients, with 16 females and 14 males. We reserved 6 patients (3 males and 3 females chosen randomly) for testing, and used the data of the other 24 patients for training. To enhance the robustness of the training process, we applied data augmentation by generating new scalograms that do not correspond to any specific patient but are labeled by class, following the procedure described in Section 3.3. In Figure 4, we present a comparison between real and generated samples, showing a random subset of real scalograms from the training set alongside synthetic scalograms produced by the cWGAN. For a quantitative assessment, we computed the Fréchet Inception Distance (FID) [118] between real and synthetic scalograms for the sex classification task, obtaining a value of 88.74. To adapt the grayscale scalograms to Inception-v3, the single channel was replicated three times and the images were resized to match the network’s input size. Although relatively high, this FID may be influenced by the domain mismatch between our grayscale scalograms and the RGB images used to pretrain Inception-v3, which can amplify the measured distance. To evaluate the impact of the amount of additional data, we performed the classification process four times by varying this amount, expressed as the percentage increase in scalograms added to the training set. The average accuracy results for sex prediction are shown in Figure 5. In this figure, we can observe that, except when increasing data by 25% or 150%, data augmentation with the cWGAN generally benefits the model’s efficiency in all other cases. Among these, optimal results appear to be achieved with a 75% data increase, where the test set window accuracy for sex prediction reaches nearly 72%. To evaluate whether the observed improvement with data augmentation is statistically significant, we repeated the classification experiment 34 times under two conditions: (i) without data augmentation, and (ii) with a 75% increase in the training set using synthetic scalograms. For each run, we computed the average test window accuracy. To compare both distributions, we applied Welch’s *t*-test, obtaining a t-statistic of −2.6169, a *p*-value of 0.0146, and a 95% confidence interval for the difference in means of [−0.1080, −0.0130]. These findings indicate that the improvement in classification accuracy due to data augmentation is statistically significant, reinforcing the hypothesis that synthetically generated data enhances the model’s ability to predict sex.

**Figure 6:**
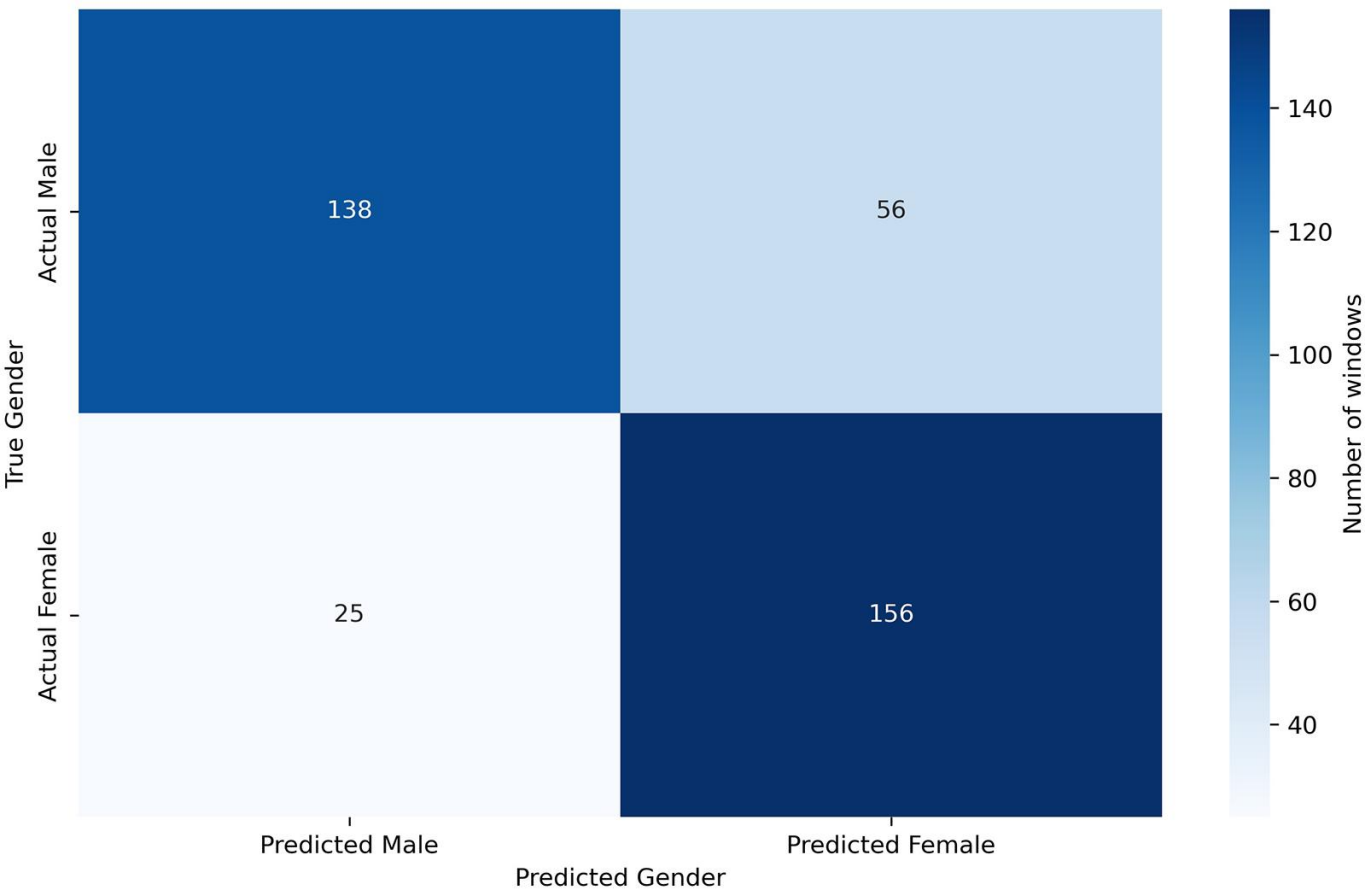
Confusion matrix for sex classification.

Given the limited number of subjects in the dataset, we can perform a leave-one-out classification for each of the 6 test patients, using the other five as additional training material. In this way, for each of these 6 patients dedicated for test, a model is trained with the remaining 29 patients plus the generated scalograms. The goal of the data augmentation process was to generate samples conditioned on specific attributes, thereby enhancing the model’s ability to control and manipulate the output based on desired characteristics. Table 3 presents the results obtained using this methodology, comparing the classification performance with only the original data to the performance with a 75% increase in data through augmentation. The application of data augmentation led to substantial improvements across all metrics. The classification accuracy for 10 s windows (i.e., after aggregating frame-level predictions into a single decision per window) rose significantly, from 72.80% to 78.40%, reflecting a 5.6% improvement. This enhancement demonstrates the model’s increased capability to accurately classify sex due to the added variability in the data. Frame-level accuracy (i.e., the performance of the CNN when predicting individual 4 s frames) also benefited, showing a 4.11% increase, which indicates a consistent trend of enhanced performance even at finer time granularities. In terms of error rates, both the FAR and FRR declined, with reductions of 6.02% and 5.69%, respectively, after applying data augmentation. Precision and recall also improved, with precision increasing by 6.02% and recall by 5.69%. These results suggest that the augmented dataset not only makes the model more accurate but also promotes a better balance between detecting true positives and minimizing false negatives. This balance is further evidenced by a 5.57% increase in the F1-score. Additionally, in Figure 6, we can observe the confusion matrix for the case where data augmentation is increased by 75%. It is important to note that we are classifying 10 s windows, which the numbers in the figure correspond to. In this figure, we can see that for the model, women are easier to identify (86.19% accuracy), whereas men present more difficulties (71.13% accuracy).

**Table 3:** Classification metrics for sex estimation with and without data augmentation.

Data	Accuracy in	Accuracy in	FAR	FRR	Precision	Recall	F1-score
augmentation	windows (%)	frames (%)	(%)	(%)	(%)	(%)	(%)
0%	72.80	64.33	26.90	27.03	73.10	72.97	72.78
75%	78.40	68.44	20.88	21.34	79.12	78.66	78.35

**Figure 7:**
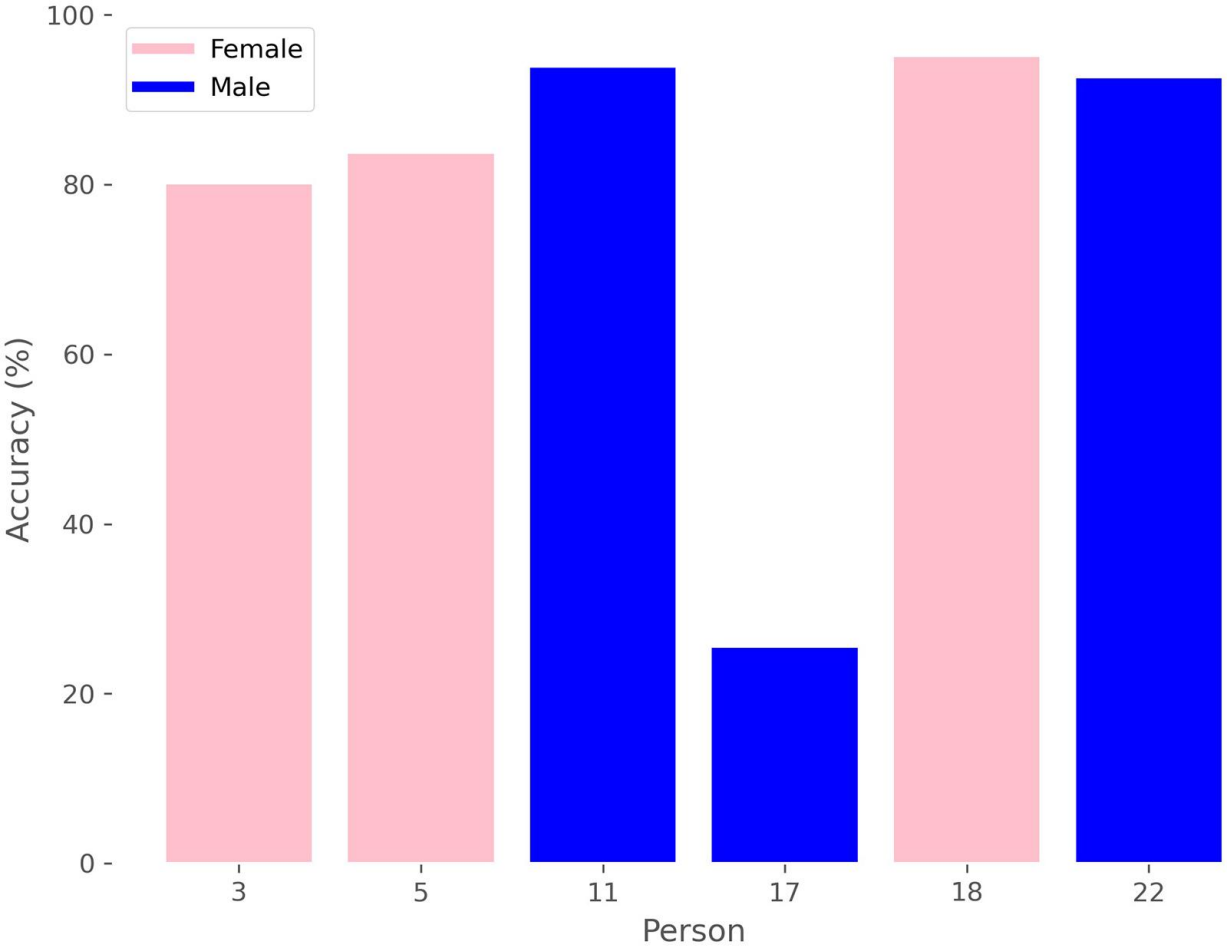
Accuracy in sex estimation by person.

Additionally, we can analyze the accuracy of sex prediction for each window in relation to the patient, as depicted in Figure 7, where we see that there is a male whose windows are mostly classified by the model as belonging to female, thereby reducing the average results.

Given the limited number of subjects in the dataset, we can perform a leave-one-out classification for each of the 6 test patients, using the other five as additional training material. In this way, for each of these 6 patients dedicated for test, a model is trained with the remaining 29 patients plus the generated scalograms. The goal of the data augmentation process was to generate samples conditioned on specific attributes, thereby enhancing the model’s ability to control and manipulate the output based on desired characteristics. Table 3 presents the results obtained using this methodology, comparing the classification performance with only the original data to the performance with a 75% increase in data through augmentation. The application of data augmentation led to substantial improvements across all metrics. The classification accuracy for 10 s windows (i.e., after aggregating frame-level predictions into a single decision per window) rose significantly, from 72.80% to 78.40%, reflecting a 5.6% improvement. This enhancement demonstrates the model’s increased capability to accurately classify sex due to the added variability in the data. Frame-level accuracy (i.e., the performance of the CNN when predicting individual 4 s frames) also benefited, showing a 4.11% increase, which indicates a consistent trend of enhanced performance even at finer time granularities. In terms of error rates, both the FAR and FRR declined, with reductions of 6.02% and 5.69%, respectively, after applying data augmentation. Precision and recall also improved, with precision increasing by 6.02% and recall by 5.69%. These results suggest that the augmented dataset not only makes the model more accurate but also promotes a better balance between detecting true positives and minimizing false negatives. This balance is further evidenced by a 5.57% increase in the F1-score. All metrics reported, except for frame-level accuracy, are computed at the window level. Additionally, in Figure 6, we can observe the confusion matrix for the case where data augmentation is increased by 75%. It is important to note that we are classifying 10 s windows, which the numbers in the figure correspond to. In this figure, we can see that for the model, women are easier to identify (86.19% accuracy), whereas men present more difficulties (71.13% accuracy).

### 4.3 Age

**Figure 8:**
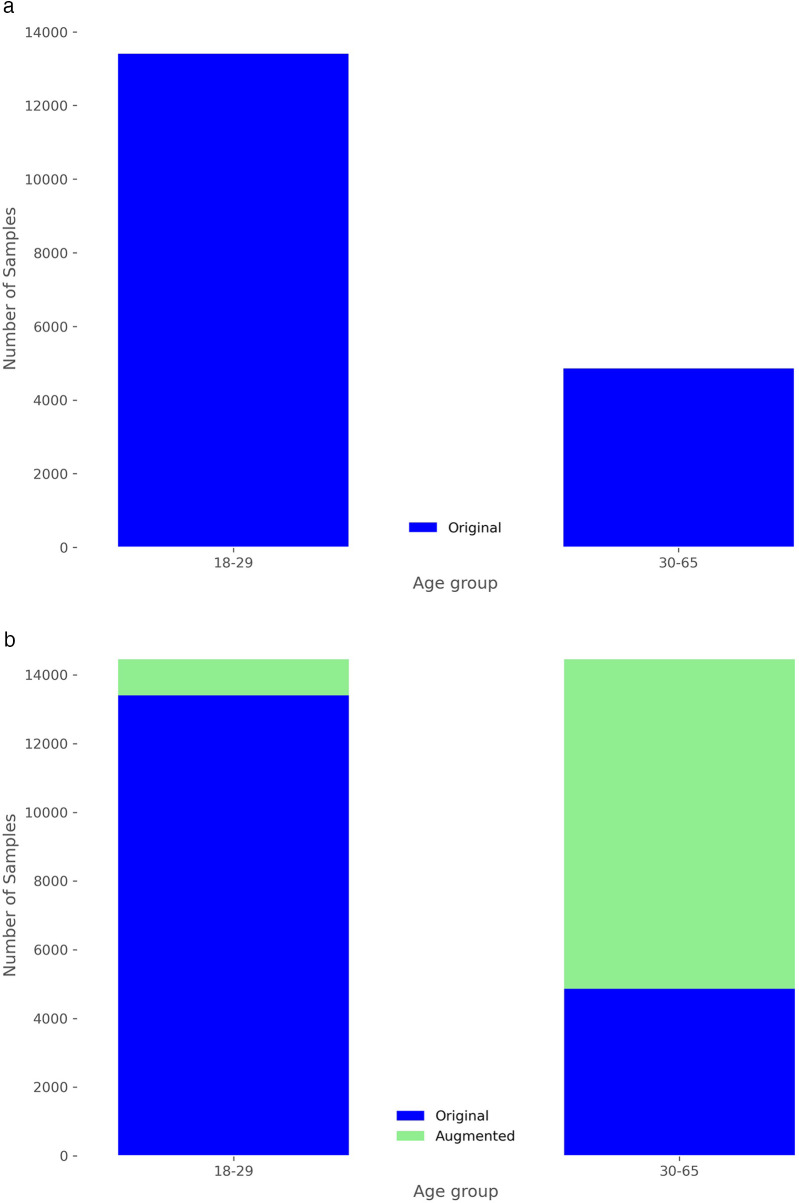
Distribution of frames in the training set by age group. **(a)** Without data augmentation. **(b)** After data augmentation of 75%.

Similarly to the sex analysis, we explored the extent to which personal information, such as age, might be conveyed through the available cardiac signals. Given the small dataset of only 30 subjects, age presents a more significant challenge, as illustrated in Figure 2b. There are age groups with no patients, and the distribution is clearly imbalanced. To mitigate this issue, we categorized the data into two groups: individuals aged 18 to 29 and those aged 30 or older. Given the imbalance in distribution, grouping them into a larger number of categories would have resulted in some groups having very few elements. Despite this grouping, the data distribution remains markedly imbalanced, as shown in Figure 8a. Therefore, data augmentation was crucial to achieve a more balanced training set. As illustrated in Figure 8b, after increasing the original dataset by 75%, the scalogram dataset for age classification became balanced across the two groups. Specifically, synthetic scalograms were generated until both classes had approximately equal sample sizes (around 14,000 each). This approach was preferred over oversampling or undersampling to preserve the original distribution and reduce the risk of overfitting. While other techniques such as SMOTE or class weighting were considered, the cWGAN was ultimately chosen due to its capacity to produce label-consistent and structurally realistic synthetic samples. In Figure 4, we also provide a comparison between real and synthetic scalograms for the age classification task. As in the sex classification analysis, we quantitatively assessed the similarity between real and synthetic scalograms for age by computing the FID, which resulted in a value of 39.56.

**Figure 9:**
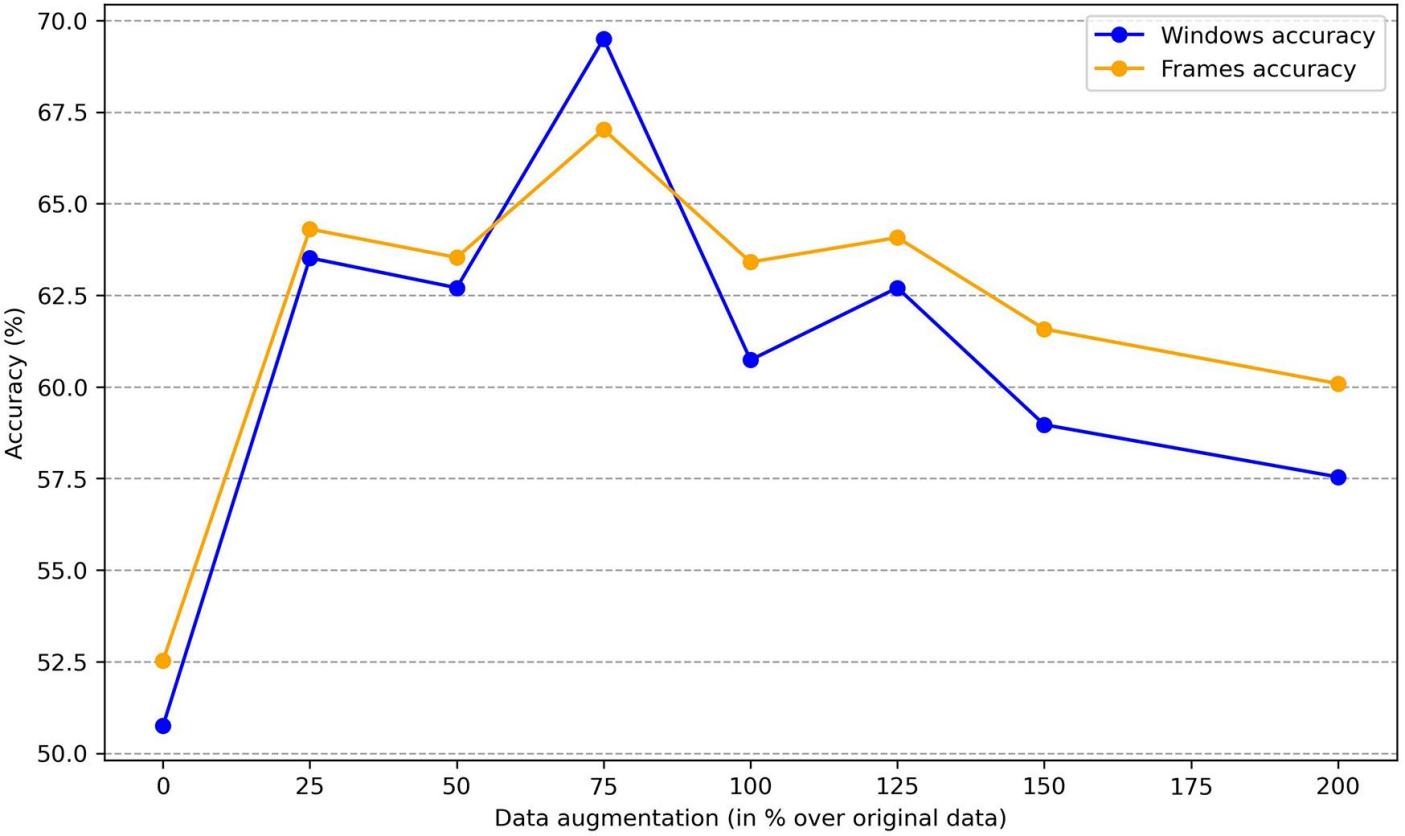
Average accuracy on age group classification by data augmentation size.

Similar to our approach with sex, we can investigate the impact of the amount of new scalograms added to the training set on the classification of test patients by age group. This analysis is illustrated in Figure 9, where the best results are obtained by training the classifier with an additional 75% of generated data. To create this plot, we performed classification four times, depicting the average results. In this figure, it is observed that the use of data augmentation does notably improve the performance of the classification algorithm between the two groups. Furthermore, the accuracy takes on a concave shape, reaching its maximum when increasing the data by 75% and decreasing thereafter. On the other hand, in Table 4, we observe the metrics comparing the results after applying data augmentation and without applying it, following the procedure described for sex using leave-one-out classification with the aim of maximizing the training data. The inclusion of 75% data augmentation resulted in a marked improvement across all classification metrics. The window-level accuracy rose from 65.60% to 72.83%, an increase of 7.23%, while frame-level accuracy similarly increased by 6.48%, reaching 69.50%. In terms of error rates, both the FAR and the FRR exhibited notable decreases, dropping from 25.73% to 18.54% and from 34.73% to 27.46%, respectively. This reduction in error rates indicates that the model becomes more reliable with data augmentation, with fewer incorrect classifications on both acceptance and rejection. Precision and recall showed notable improvements, with increases of 7.19% and 7.27%, respectively, compared to the initial values. The F1-score also rose by 8.85%, reflecting a better balance between precision and recall. As in the case of sex, we evaluated the statistical significance of the observed improvement by repeating the classification experiment 34 times and applying Welch’s *t*-test. The results yielded a t-statistic of −2.4017, a *p*-value of 0.0225, and a 95% confidence interval of [−0.1067, −0.0087], indicating that the performance gain from data augmentation is statistically significant in the age group classification task.

**Table 4:** Classification metrics for age group classification with and without data augmentation.

Data	Accuracy in	Accuracy in	FAR	FRR	Precision	Recall	F1-score
augmentation	windows (%)	frames (%)	(%)	(%)	(%)	(%)	(%)
0%	65.60	63.02	25.73	34.73	74.27	65.27	61.81
75%	72.83	69.50	18.54	27.46	81.46	72.54	70.66

**Figure 10:**
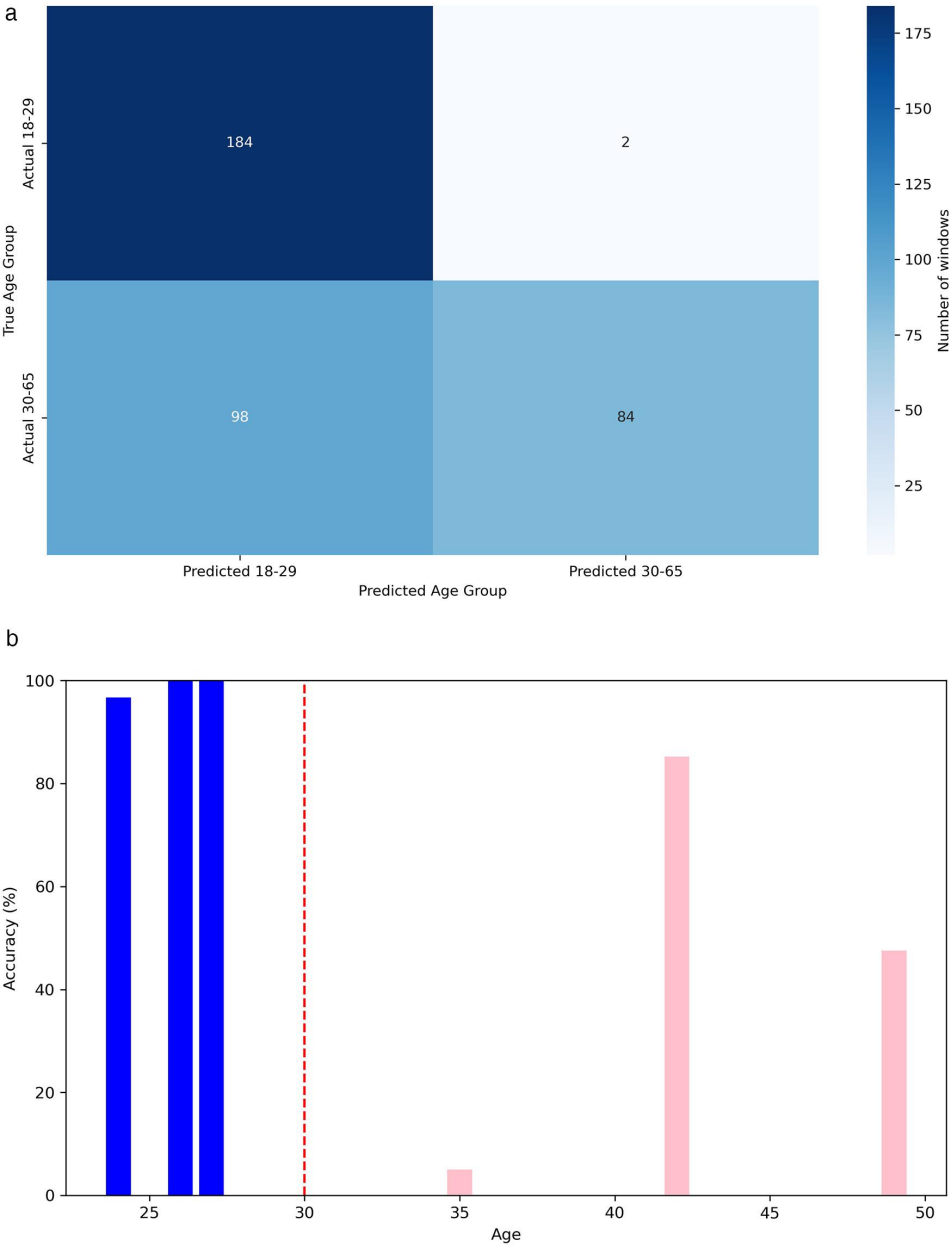
Performance metrics for age group classification. **(a)** Confusion matrix for classification in age group. **(b)** Accuracy in age group classification by person.

In Figures 10a, 10b, we can respectively observe the confusion matrix and the accuracy when predicting the age group for each person’s windows, ordered by age. In Figure 10a, we observe that the model exhibits a slight bias, tending to classify a higher number of windows into the most frequent age group in the dataset, which is 18 to 29 years. Additionally, one of the six individuals in the test set, a 35-year-old woman, is being classified as under 30. It is worth clarifying that the age detected through heart signals does not uniquely correspond to a person’s chronological age but rather expresses the general state of the cardiovascular system [12]. In certain cases, this may cause individuals with ages near the boundaries of age groups to be misclassified. This error might not be attributed to the model in some cases, but rather to the characteristics and condition of their particular cardiovascular system.

### 4.4 Explainability

Despite the strong performance often achieved by deep learning models, their black-box nature can limit interpretability and hinder trust in their outputs. In our context, where cardiac radar signals are analyzed using convolutional neural networks (CNNs) to infer demographic attributes, understanding the rationale behind the model’s decisions is crucial—both for scientific insight and for fostering confidence in the system. To this end, we employed Gradient-weighted Class Activation Mapping (Grad-CAM) [119] to generate visual explanations of the CNN’s predictions. Grad-CAM produces heatmaps that highlight the regions of the input scalogram that most strongly influence the output, enabling the identification of time-frequency patterns the model deems most relevant for distinguishing between classes such as sex or age group. Incorporating such explainability not only improves the transparency of our system but also provides a means for qualitative assessment and validation of the model’s behavior.

**Figure 11:**
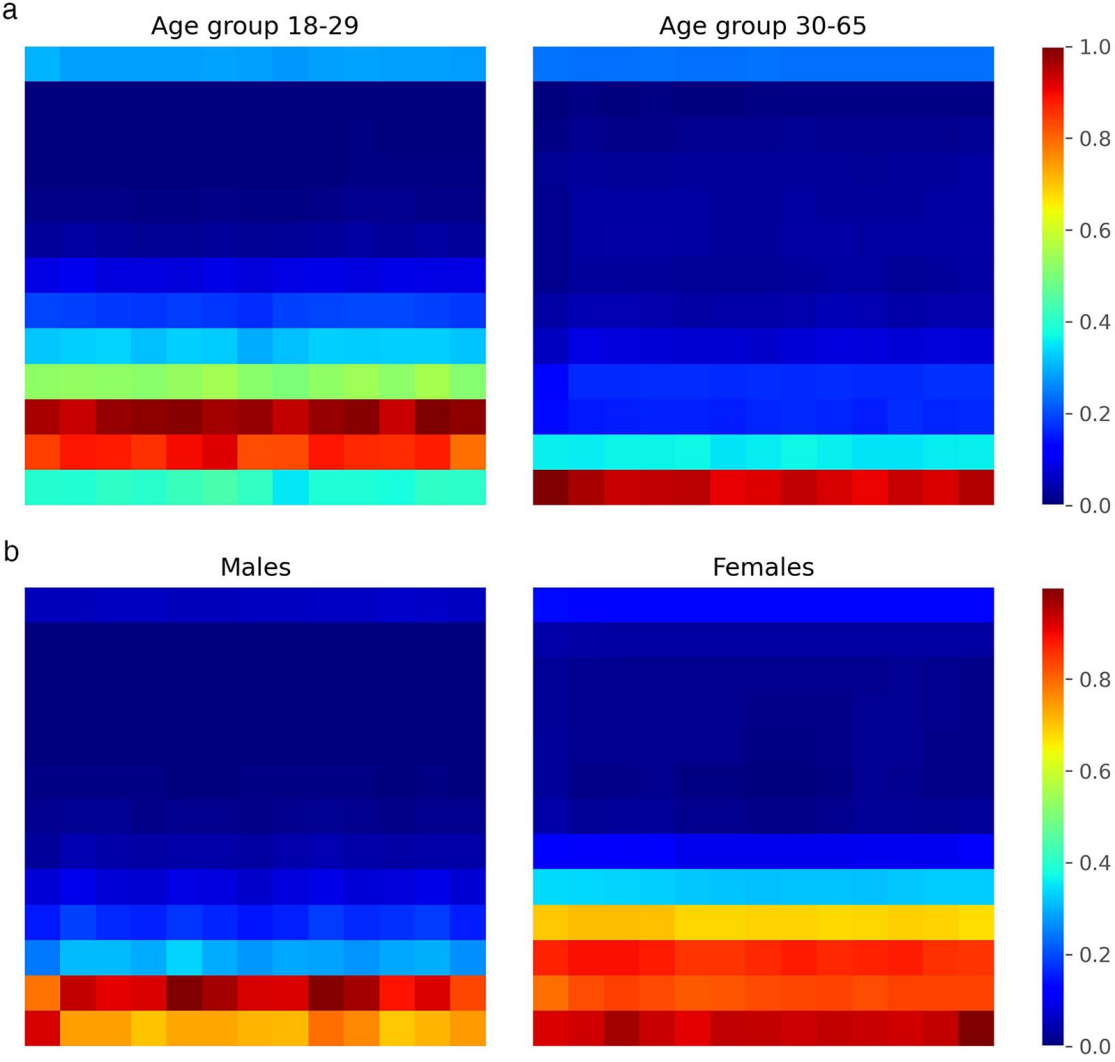
Grad-CAM heatmaps showing CNN attention regions on scalograms. **(a)** Age group classification. **(b)** Sex clasification.

To analyze the internal decision-making process of our CNN classifier, we integrated Grad-CAM into the fourth convolutional layer of the network. We trained the CNN multiple times (five repetitions) with the augmented dataset to ensure robustness, and then applied Grad-CAM to the test set to identify the regions of the input scalograms that most influenced the model’s predictions. For each correctly classified instance, we extracted class-specific activation maps and aggregated them separately for each class (male and female). This yielded averaged heatmaps representing the most salient time-frequency patterns used by the network to distinguish between the two categories. The results for both sex and age classification tasks are presented in Figure 11. In general, the network consistently allocates more attention to the low-frequency regions of the scalogram, which appear to contain the most discriminative information for class separation, while higher frequencies receive comparatively less focus. This observation suggests that future work could benefit from emphasizing this lower frequency range, which seems to play a decisive role in the model’s decisions. In both classification tasks, we also observe class-specific differences in the regions of highest activation. These differences are particularly pronounced in the sex classification task, where the network tends to focus on slightly wider frequency bands for female subjects. As previously suggested in the literature [81], this might be attributed to morphological differences in the chest between male and female individuals, which could influence the cardiac radar signal and, consequently, the frequency content of the resulting scalograms. Similarly, in the age classification task, differences in activation patterns may reflect known physiological changes in cardiac dynamics with age. For instance, cardiac features such as Heart Rate Variability (HRV) have been shown to vary with age [120], which may contribute to the distinct attention patterns observed between the two age groups.

### 4.5 Use of transfer learning

Due to the limited number of patients in our dataset, Transfer Learning is one of the most common approaches to improve model training in scenarios with scarce data [121]. Transfer Learning is particularly valuable in cases with restricted data availability, as it transfers relevant representations from previously trained models, optimizing the training process and potentially enhancing prediction accuracy. Moreover, its applicability extends to various biomedical tasks where acquiring large volumes of labeled data is both costly and challenging [122].

In our study, we evaluated four different pre-trained networks by applying fine-tuning to each. This *fine-tuning* process involves freezing the parameters of the network’s initial layers, thereby retaining the general features learned from a large and diverse dataset, while training only the final layers to adapt to the specific characteristics of our study. The networks evaluated included ResNet50 [123], DenseNet [124], ShuffleNet [125], and VGG-19 [126], all pre-trained on large-scale, real-world image datasets. Despite the potential advantages, the performance results achieved with these networks were not satisfactory. Tables 5, 6 show the classification results by sex and age group using each of these networks with 75% data augmentation applied consistently across all cases.

**Table 5:** Sex classification metrics with different pre-trained networks, using 75% of data augmentation.

Network	Accuracy in	Accuracy in	FAR	FRR	Precision	Recall	F1-score
	windows (%)	frames (%)	(%)	(%)	(%)	(%)	(%)
ResNet-50	49.07	49.84	51.26	51.23	48.74	48.77	48.54
DenseNet	56.27	60.20	43.85	43.99	56.15	56.01	55.88
ShuffleNet	45.87	50.49	54.26	54.24	45.74	45.76	45.74
VGG-19	54.93	55.53	44.93	44.95	55.07	55.05	54.92
Our network	78.40	68.44	20.88	21.34	79.12	78.66	78.35

**Table 6:** Age group classification metrics with different pre-trained networks, using 75% of data augmentation.

Network	Accuracy in	Accuracy in	FAR	FRR	Precision	Recall	F1-score
	windows (%)	frames (%)	(%)	(%)	(%)	(%)	(%)
ResNet-50	55.98	53.83	43.13	44.24	56.87	55.76	54.02
DenseNet	47.55	49.43	54.26	52.77	45.74	47.23	42.31
ShuffleNet	45.65	46.81	54.63	54.45	45.37	45.55	45.07
VGG-19	58.15	59.35	38.85	42.14	61.15	57.86	54.67
Our network	72.83	69.50	18.54	27.46	81.46	72.54	70.66

The performance of transfer learning models such as ResNet-50, DenseNet, ShuffleNet, and VGG-19 was noticeably lower than that of our custom-designed network. We attribute this performance gap primarily to domain mismatch [127]: these architectures are pre-trained on ImageNet, a dataset of natural images with vastly different structural and spectral characteristics compared to radar-derived cardiac scalograms. While transfer learning is effective when source and target domains are similar, in this case, the feature representations learned from natural textures, shapes, and objects do not transfer well to time-frequency biomedical signals. We also experimented with fine-tuning deeper layers and adding domain-specific dense layers on top of the pre-trained backbones. However, these adjustments did not yield substantial improvements, suggesting that the core convolutional filters learned from natural images were suboptimal for this task. These results indicate that task-specific architecture design and training from scratch are more effective strategies for modeling radar-acquired physiological signals.

### 4.6 Evaluating threat feasibility through temporal aggregation

**Figure 12:**
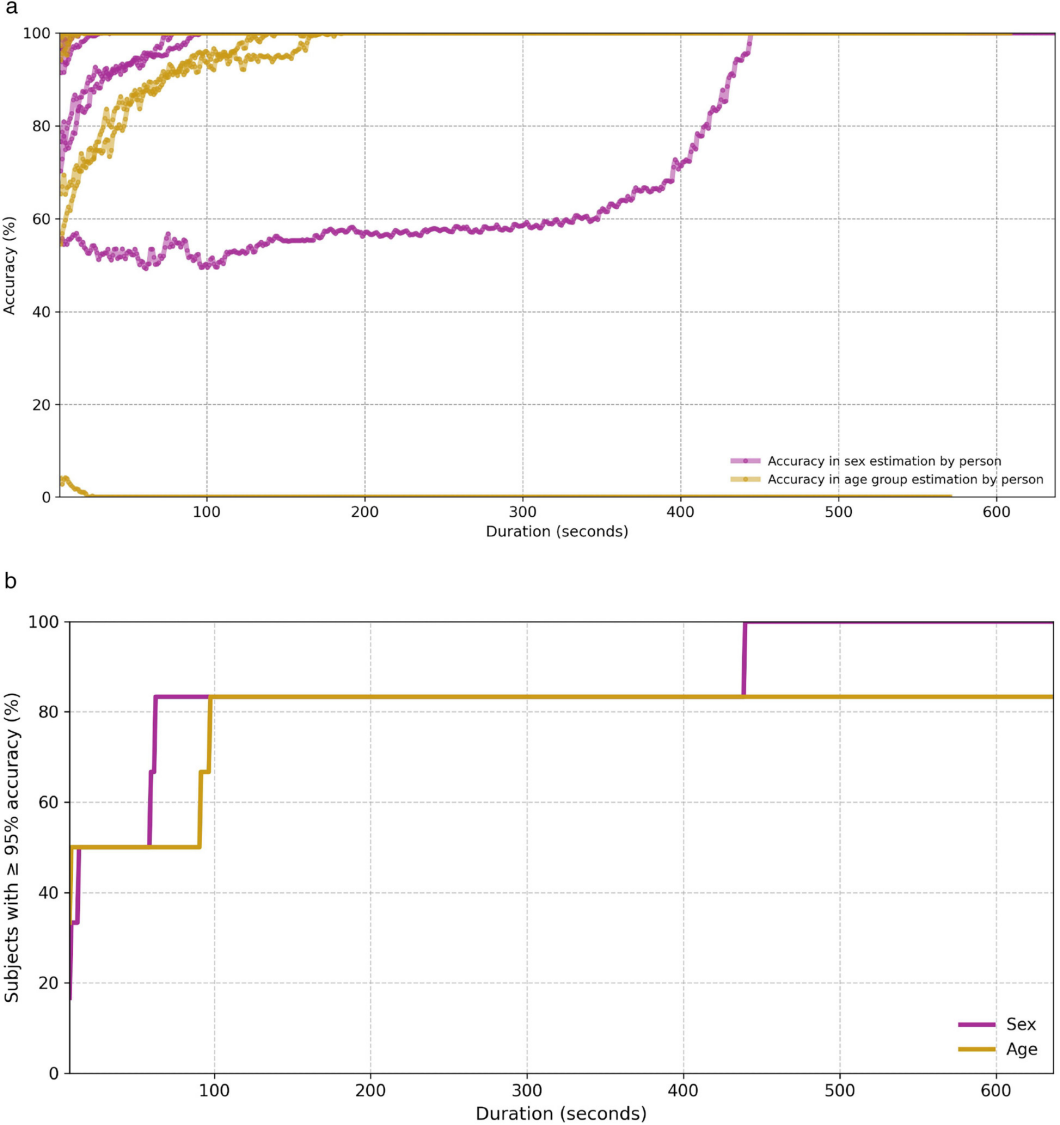
Classification accuracy for both sex and age group as a function of temporal aggregation. **(a)** Average classification accuracy per test subject over temporally aggregated subsets of varying duration. **(b)** Cumulative percentage of test subjects for whom the model achieves at least 95% accuracy, as a function of temporal input duration.

To assess the practical feasibility of the privacy threat described in Section 3.5, we conducted an additional experiment based on the hypothesis that an adversary could improve the reliability of demographic inference by aggregating predictions over longer temporal spans. Specifically, we evaluated the classification accuracy of our model when predictions are aggregated from multiple consecutive input segments corresponding to the same individual. Since the model provides a prediction for each individual frame, we generated all possible contiguous subsets of frames for each subject in the test set (6 subjects), where each subset spans at least 7 seconds. For each subset, a final prediction was obtained by applying majority voting over the model’s predictions for the individual frames within that subset. We then grouped these subsets into bins according to their temporal length and computed the average accuracy for each bin. The results are shown in Figure 12a, where each line corresponds to a single test subject and each point represents the average accuracy over all subsets of that specific duration. As can be observed, in the age group classification task, one subject—a 35-year-old female—is consistently misclassified as belonging to the 18–29 age group, regardless of the temporal span. Aside from this case, the overall accuracy increases steadily as the input duration grows, supporting the notion that temporal aggregation significantly boosts inference reliability. In the age classification task, for example, three out of the six test subjects reach 100% accuracy when the aggregated input covers at least 20 s. Figure 12b complements this analysis by showing the cumulative percentage of subjects for whom the model achieves at least 95% accuracy at or before a given input duration. Notably, for both tasks, the model predicts the correct class with high confidence (≥95%) for 80% of subjects after observing less than 100 s of data. These findings provide empirical support for the proposed privacy threat model. By demonstrating that demographic attributes such as sex and age can be inferred with high confidence from temporally aggregated cardiac radar signals—even in the absence of physical contact or subject cooperation—our results highlight the tangible risks of long-term passive monitoring. The consistent increase in prediction accuracy with longer input durations suggests that an adversary with access to sustained radar observations could reliably construct demographic profiles of individuals over time.

## 5 Discussion & conclusions

The results of this research suggest that radar-extracted cardiac signals, while primarily intended to capture relevant medical features, also inadvertently encode personal information such as sex and age, which could pose privacy concerns. This characteristic, also observed in other biometric data types like facial recognition and ECG, highlights the importance of implementing robust security measures for the storage and management of such data. Specifically, we achieved an accuracy of 78.40% for correctly predicting sex, and 72.83% for predicting the patient’s age group, consistently using 10 s temporal windows. To accomplish this, we employed a model primarily based on a CNN that classifies the scalograms derived from cardiac signals into their respective categories. An important tool for improving these results was data augmentation, which we conducted using a cWGAN, also studying the amount of extra data that most positively influences the model’s performance. Both for sex (Figure 5) and for age (Figure 9), experiments determined that a 75% increase in the original training set offered optimal performance. This increase in accuracy was 5.6% in sex estimation and 7.23% for age (Tables 3, 4). On the other hand, the use of pre-trained networks on large image datasets (Transfer Learning) did not prove beneficial in our case, yielding results that were inferior to those of our own model, which was trained solely on samples obtained from the original dataset. Hyperparameter optimization for both the CNN classifier and the cWGAN model was not performed and remains an open task for future work. Due to the limited size of the dataset, we did not perform a full grid search. Instead, hyperparameters were selected through manual tuning informed by prior literature and validation performance. Future work will include a systematic search strategy, such as Bayesian optimization or nested cross-validation, to improve reproducibility and fairness.

However, while traditional methods such as ECG benefit from larger datasets and have shown high performance in demographic attribute estimation, they still face challenges regarding portability, comfort, and suitability for continuous monitoring. In contrast, radar-based techniques offer a good contactless alternative but are currently limited by smaller datasets, which can affect both model accuracy and generalization capacity. Additionally, due to the novelty of this approach, further studies are required to systematically evaluate its robustness in diverse real-world settings. This comparison can be seen in Table 7, where we compare our results with those obtained through other signals, as we are not aware of previous studies conducting this procedure with this type of signal. In this table, it is observed that the accuracy in estimating sex usually exceeds 90%, and the databases used sometimes include hundreds of thousands of patients.

**Table 7:** Summary of studies on Age and Sex estimation using biosignals.

Ref (Year)	Type	Biosignal	Results	Comments	Patients
Attia et al. [72]	Age & Sex	ECG	Sex: 90.4% accuracy, Age: average error of 6.9 ± 5.6 years ($R^{2}=0.7$)	CNN	774,783
Kaushik et al. [130]	Age & Sex	EEG	Sex: Accuracy of 93.7%, Age: Accuracy 97.5% (divided in 6 age groups)	Bi-directional LSTM and LSTM	60
Lyle et al. [131]	Sex	ECG	Dataset1: accuracy 91.3%, Dataset2: accuracy 86.3%	Symmetric Projection Attractor Reconstruction method	104 8,903
Saho et al. [80]	Sex	Radar-based gait features	Accuracy of 93.6%	Multilayer Perceptron	181
Lima et al. [132]	Age	ECG	MAE: 8.38 ± 7.00 (CODE), 8.44 ± 6.19 (ELSA-Brasil), 10.04 ± 7.76 (SaMi-Trop)	Deep neural network	1,558,415 14,236 1,631
Cabra Lopez et al. [73]	Sex	ECG	Accuracy 94.4% ± 2.0%	GoogLeNet	202
Wang et al. [78]	Sex	Radar-based body movement	Accuracy of 90%	Analyzes transition between sitting and standing. CNN	178 elderly people
van der Wall et al. [76]	Age	ECG	Average error of 6.9 ± 5.6 years ($R^{2}=0.72\pm 0.04$)	Neural network regression model	6,228 healthy subjects
Chang et al. [77]	Age	ECG	MAE 6.899 years (r = 0.822)	CNN with attention mechanism for potassium concentration estimation	71,741
Adib et al. [133]	Age	ECG	MAE: 3.99 (all subjects), 2.99 (healthy subjects)	Deep neural network model	4,884
Cabra Lopez et al. [134]	Sex	ECG	Accuracy 94.82% ± 1.96%	DCNN, RGB wavelet transformation	202
Kaneko et al. [135]	Age	ECG	Accuracy 68.19%	They analyze Heart Rate Variability, classifing in two age groups with Random Forest	420,000
Naser et al. [136]	Sex	ECG & hormones	ECG could be a biomarker of hormone status	Artificial intelligence-augmented ECG and sex hormone levels	90,337
Dias et al. [137]	Age & Sex	ECG	Sex: F1-score 0.800 ± 0.007; Age: MAE 8.961 ± 0.180	ResNeXt-based architecture trained with CODE15 dataset	1,558,415
Wei et al. [138]	Age & Sex	EEG	Sex: 55.07% accuracy; Age: 66.67% accuracy (allowing 1 year error)	Random Forest	351 children
Niu et al. [139]	Sex	EEG	Accuracy of 95.2% accuracy	k-means clustering and SVM	232
Our proposal (2024)	Age & Sex	Radar-based cardiac signal	Sex: Accuracy of 78.40%, Age: Accuracy of 72.83%	Data augmentation with GAN, CNN, 2 age groups	30

Overall, the small dataset size can impose certain limitations on the generalization of these results, as dedicating only 6 patients for testing means that the incorrect prediction of just one of them can significantly influence the final results. This is evident in both sex and age cases, where in each, there is a subject consistently misclassified into the wrong class. In the case of sex, this occurs with patient 17 (Figure 7), who is the male with the lowest body weight in the dataset (28 years, 165 cm, 57 kg), while for age, it occurs with a 35-year-old woman, who is near the boundary between both age groups. Furthermore, public datasets for this type of signal remain very limited, with 30 patients representing a number that is actually higher than the average found in other studies [105]. It is also important to note that we are dividing the training set and the test set by patients, ensuring that the model has never seen data from the same patient when predicting the class of a window. This procedure brings the study’s results closer to potential real-world applications.

Our results show that classification accuracy improves significantly when predictions are aggregated over longer temporal windows, indicating that even models with moderate single-frame performance can achieve high reliability given sustained observations. These findings highlight the potential for passive adversaries to infer sensitive demographic attributes through unobtrusive, long-term data collection, emphasizing the need for robust privacy protections. Our concerns align with biometric information protection standards such as ISO/IEC 24745 [128], the General Data Protection Regulation (GDPR) [9], and NIST Special Publication 800-63 [129], which require safeguarding against unauthorized identity disclosure and the inference of secondary attributes without explicit consent. This work underscores the necessity to develop effective countermeasures and privacy-preserving techniques for radar-based cardiac monitoring systems. To mitigate these risks, future work should explore:

- Signal sanitization techniques, such as adversarial perturbations or autoencoder-based anonymization, to suppress demographic features while preserving the primary utility of the signal.
- Differential privacy mechanisms, applied at the feature extraction or model output level, to provide formal privacy guarantees against attribute inference.
- Detection and auditing frameworks capable of identifying unauthorized use of inference models or repeated inference attempts in deployed systems.

As a conclusion of our study, we can affirm the presence of personal information within radar-extracted cardiac signals that could be exploited for identification purposes. As a result, it is crucial to treat these signals with the same level of security and privacy considerations as more traditional biometric data. This is particularly important in the context of biomedical applications, where the misuse of personal health information could have serious ethical and legal implications. A specific example of how data leakage can compromise the security of widely used applications is the use of sex and age information in e-passports. This information is utilized to derive the access key for the RFID chip embedded in the passport, a practice that has been in effect since September 11 [11]. Future research should focus on improving the accuracy and robustness of these methods but also on developing protocols and technologies to safeguard the collected data. By addressing these concerns, we can harness the potential of radar-based cardiac signal analysis for a wide range of medical and healthcare applications while maintaining the trust and confidence of the public.

Therefore, non-invasive monitoring methods, such as radar-based cardiac signal analysis, offer substantial benefits in healthcare by enabling continuous monitoring without the need for direct contact with the patient. This technology is particularly valuable in scenarios where patient mobility or frequent follow-up is challenging, as it provides an efficient, unobtrusive way to track vital signs and other health metrics over time. However, this accessibility also introduces privacy risks, as sensitive biometric information can be derived from these signals, including details about a patient’s sex, age, and possibly unique identifying characteristics. If improperly safeguarded, this information could be exploited in unintended ways, posing risks to patient privacy and autonomy. The trade-off lies in balancing the advantages of improved patient monitoring and personalized care with stringent data protection measures to prevent unauthorized access and misuse of personal health information. Ultimately, ensuring the security of radar-based health data will be essential for achieving patient trust, regulatory compliance, and ethical application in healthcare.

## Data Availability

The authors affirm that this study utilized a pre-existing, publicly available dataset sourced from a previous publication. Specifically, the data employed in this research were obtained from “A dataset of clinically recorded radar vital signs with synchronised reference sensor signals”, originally published by Sven Schellenberger, Kilin Shi, Tobias Steigleder, Anke Malessa, Fabian Michler, Laura Hameyer, Nina Neumann, Fabian Lurz, Robert Weigel, Christoph Ostgathe, and Alexander Koelpin in Scientific Data (2020). Our use of this dataset adheres to its Creative Commons Attribution 4.0 International (CC BY 4.0) license. Full and proper citation of the original source is provided in the References section of this article.
